# Brain metastasis secondary to primary vaginal clear cell adenocarcinoma not associated with diethylstilbestrol: a case report and a review of the literature

**DOI:** 10.3389/fonc.2026.1853333

**Published:** 2026-09-03

**Authors:** Esther Anyimadu Buckson, Obi Emereole, John Richard Ouma

**Affiliations:** 1 Department of Neurosurgery, Chris Hani Baragwanath Academic Hospital, Gauteng, Johannesburg, South Africa; 2 Neurosurgery, Awryp Medical Center, Johannesburg, South Africa; 3 Neurosurgery, Wits University Donald Gordon Medical Center, Johannesburg, South Africa

**Keywords:** case report, cerebral metastases, non-DES vaginal clear cell adenocarcinoma, PCCAV, rare

## Abstract

**Background:**

Cases of primary clear cell adenocarcinoma of the vagina (PCCAV) are infrequently encountered and account for only 5%–10% of all primary vaginal cancers 1. The most common associated risk factor for developing PCCAV is *in utero* exposure to diethylstilbestrol (DES). Patients with non-DES-related PCCAV are even scarcer and usually have a worse prognosis. PCCAV has been reported to metastasize to the lungs, supraclavicular lymph nodes, and the pelvis, while metastases to the liver, brain, and peritoneum are extremely rare. We present a rare case of a 17-year- old female patient with cerebral metastases from a non-DES-related PCCAV.

**Case report:**

A 17-year-old female patient presented to the medical emergency unit at the Chris Hani Baragwanath Academic Hospital with a 2-month history of left-sided weakness, complicated by a 2-week history of generalized tonic–clonic seizures, vomiting, and headaches. Three months before this presentation, she had been experiencing menorrhagia associated with foul vaginal discharge, which was unresponsive to treatment received at peripheral hospitals. Further examination by the gynecology team revealed a vaginal mass which was subsequently biopsied. A CT scan was performed as part of her workup, and the results showed a right contrast-enhancing parietal mass with extensive vasogenic edema, causing significant midline shift.

**Results:**

The histology from the vaginal mass favored a clear cell vaginal adenocarcinoma, while that from the parietal mass was determined to be a metastatic clear cell vaginal adenocarcinoma. Pulmonary metastases were also noted on CT staging.

**Conclusion:**

Cerebral metastases from non-DES-related vaginal clear cell tumor are extremely rare. Our case adds to the existing body of knowledge, highlights the role of neurosurgery in its management, and demonstrates the need for the development of relevant standard management guidelines.

## Graphical Abstract

**Figure:**
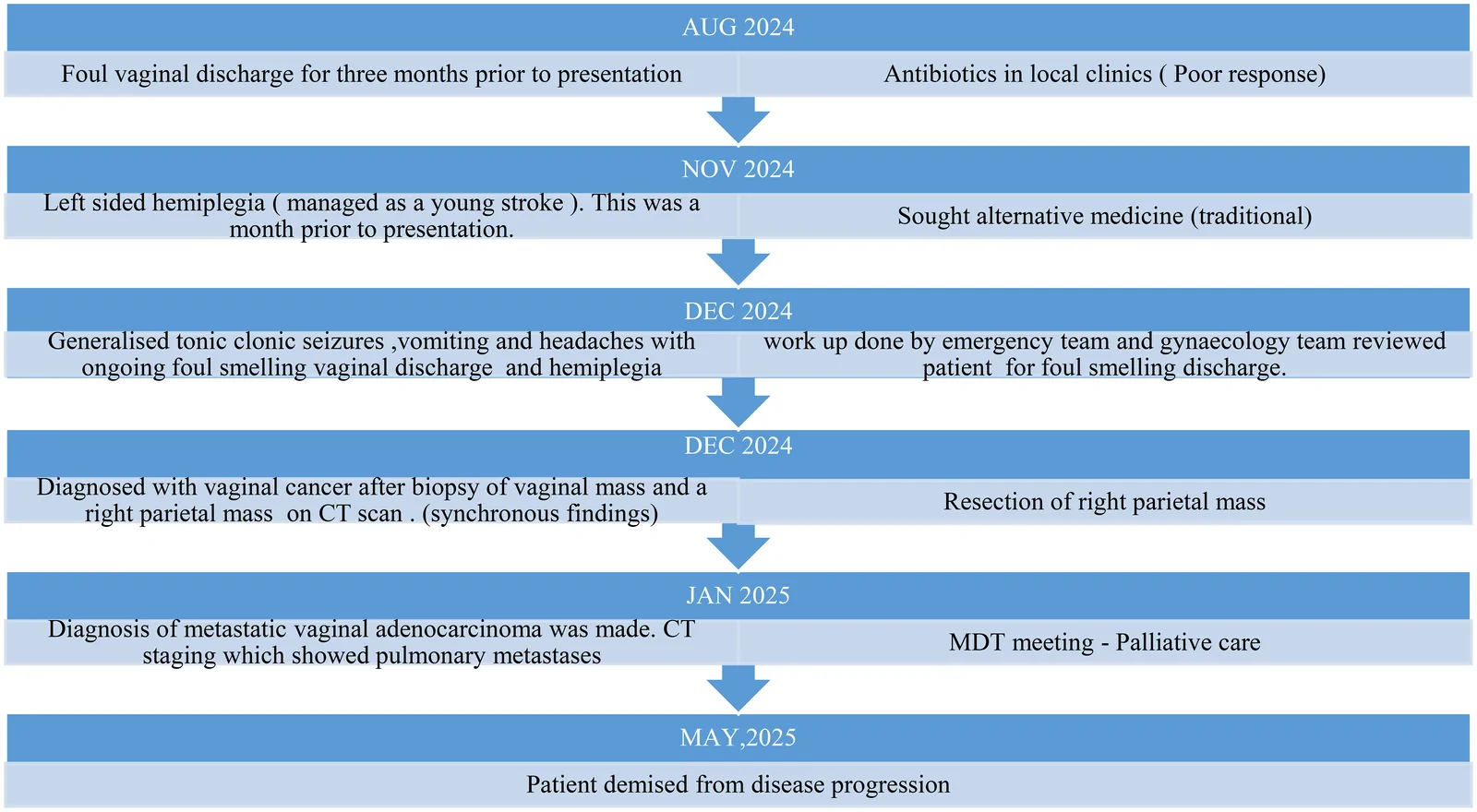
Timeline of clinical events.

## Introduction

Primary vaginal cancer accounts for 1.4% of all gynecological malignancies (1), with the majority being vaginal squamous cell carcinomas. Primary clear cell adenocarcinoma of the vagina (PCCAV) is very rare and represents 5%–10% of all primary vaginal cancers (2). To date, the most common confirmed risk factor associated with developing PCCAV is *in utero* exposure to diethylstilbestrol (DES) (3, 4). DES is a drug that was used in the 1940’s to prevent miscarriages. However, there are also cases of PCCAV where *in utero* exposure to DES is absent, and these represent an even rarer entity with only a few reported cases (4–6). PCCAV without *in utero* exposure to DES carries a poorer prognosis, response to adjuvant therapy (7), and a higher frequency of distant metastases, especially to the lungs (8). PCCAV has been reported to metastasize to the lungs, supraclavicular lymph nodes, and the pelvis, but metastases to the liver, brain, and peritoneum are less frequent (2). The development of PCCAV in those exposed *in utero* to DES through to the age of 24 has been reported to be as high as 1.4 per 1,000 (9). However, there is a second group who presents with PCCAV around the time of menopause (10), and the majority of these patients have no record of *in utero* exposure to DES (10). In 2007, there was a published case of DES-associated PCCAV in a middle-aged woman with metastasis to the brain after initial therapy (11). In 2021, Chatterjee et al. presented an up-to-date literature review of PCCAV (**Table 1**), and none had reported brain metastases (5). We present a rare case of a 17-year-old female patient with no history of sexual activity, no *in utero* exposure to DES, no genitourinary abnormalities, and negative serology for retroviral disease who presented with cerebral metastases from PCCAV.

**Table 1:** Literature review on metastatic clear cell adenocarcinoma of the cervix or vagina.

Number	Author	Age(years)	DES exposure	Location ofclearcelladenocarcinoma	Metastasis tothebrain(location)	Metastasis toothersites
1.	Burks et al., 1990 (12)	Not declared	Yes	Cervix	Yes (cerebellum)	Lung
2.	Nesme et al., 1993 (13)	27	Yes	Vagina	No	Lung
3.	Watanabe et al., 2000 (14)	63	No	Vagina	No	Lung Liver Para-aortic lymph nodes
4.	Lin et al. 2007 (11)	43	Yes	Vagina	Yes (Frontal lobe)	No
5.	Alipour et al. 2008 (15)	8.6	No	Vagina	No	Lung
6.	Nomura et al., 2015 (8)	42	No	Vagina	No	Para-aortic lymph node
7.	Lei et al., 2024 (16)	40	No	Vagina	No	Lung

## Case report

**Figure 1:**
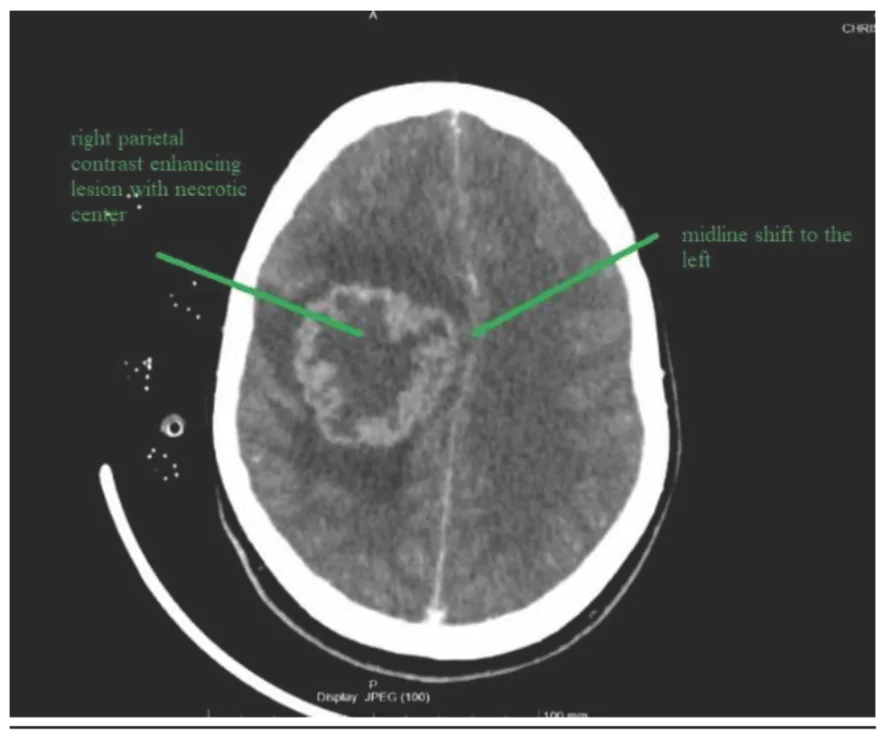
Axial CT scan showing a heterogeneously enhancing mass with central necrosis and surrounding edema in the right parietal region.

**Figure 2:**
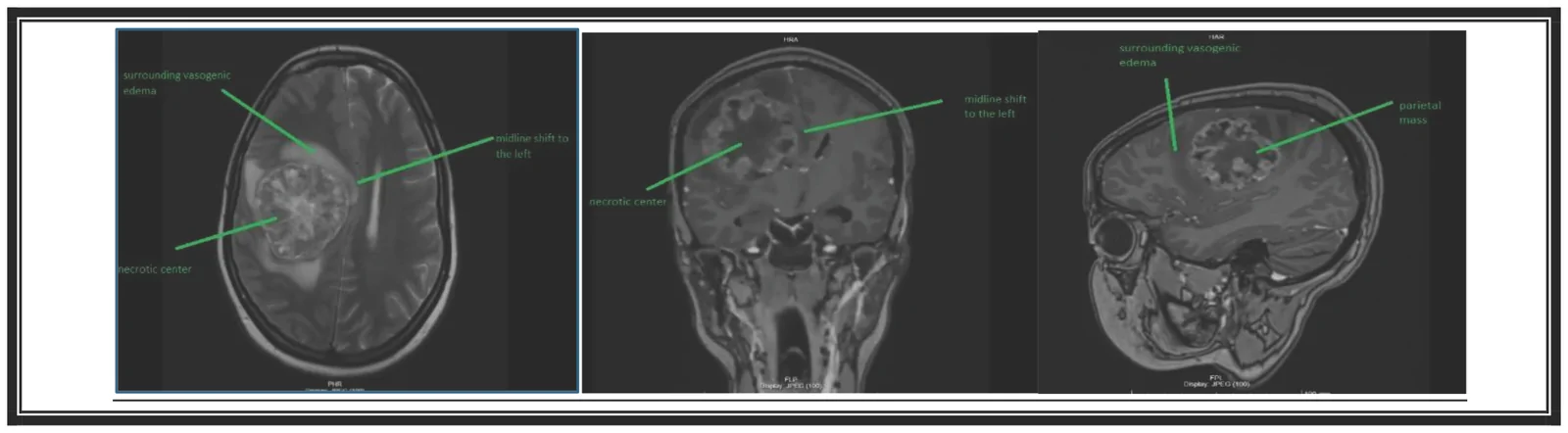
MRI: **(A)** axial T2 imaging, **(B)** coronal T1 with contrast imaging, and **(C)** sagittal T1 with contrast showing a heterogeneously enhancing mass with surrounding edema causing midline shift to the left.

A 17-year-old female patient, with no history sexual activity, no *in utero* exposure to DES, and no known comorbidities presented with a 2-month history of left-sided weakness, which was initially managed as a young stroke in peripheral hospitals. This left hemiparesis became complicated with a 2-week history of generalized tonic–clonic seizures, vomiting, and headaches, which facilitated her presentation to the hospital. A CT scan of the brain was ordered on presentation as part of her workup to establish a diagnosis. On further engagement, the patient had been experiencing a foul-smelling vaginal discharge for the past 3 months, which had not responded to antibiotics prescribed by local clinics. Her menarche was at 14 years, and for the past year, she had also been experiencing menorrhagia. On account of these findings, the gynecology team was consulted, and their examination revealed a vaginal mass that was biopsied. The results from this biopsy confirmed a clear cell vaginal adenocarcinoma. The CT scan performed by the emergency unit on presentation also showed a heterogeneous contrast-enhancing right paraventricular parietal mass with extensive vasogenic edema causing significant midline shift (**Figure 1**). MRI was also performed (**Figure 2**) to assist in surgical planning. The intracranial mass was surgically accessible, and the patient was symptomatic from raised intracranial pressure; as such, the decision was made to perform an emergent gross total resection of the brain mass, followed by management of the vaginal mass by the gynecological team. Histopathology confirmed metastasis from clear cell vaginal adenocarcinoma, making it a International Federation of Gynecology and Obstetrics (FIGO_ stage IVB. CT staging confirmed pulmonary metastases, and the patient was deemed to have oligometastatic PCCAV, with synchronous metastases at presentation.

### Postoperative management

The patient’s symptoms of raised intracranial pressure and seizures resolved, but the left hemiparesis persisted.

Histology: Sections show lesional tissue comprising a neoplastic infiltrate arranged in solid sheets and cords. The individual cells display abundant, clear-to-pale eosinophilic cytoplasm with distinct borders. Extensive pleomorphism with tumor giant cells is present. Brisk mitotic activity, including atypical forms, is noted. There is extensive background necrosis.

Immunohistochemistry: Immunoperoxidase staining for Napsin A was performed, and the controls were positive.

The patient’s case was discussed in a multidisciplinary team (MDT) meeting that included a radiation and medical oncologist, and the decision was made to offer palliative care on account of the advanced stage of the disease. The gynecological team then decided against resection on account of the poor prognosis. The patient died within 5 months of diagnosis due to disease progression. There are currently no guidelines for the management of metastatic PCCAV, and therefore, the management of cases is based on decisions made by the treating team. This case is a clear testament to this gap in the management of these cases and highlights the need for standard treatment guidelines for metastatic PCCAV.

## Discussion

PCCAV is uncommon and has been often associated with *in utero* exposure to DES. Women born between 1951 and 1953, a period when DES use for threatened abortion was at its peak, have higher incidences of clear cell adenocarcinoma (CCA) than women from any other comparable period of time (2). The risk of developing CCA among women exposed to DES through the age of 24 years has been reported to be as high as 1.4 per 1,000 (8).

The pathogenesis by which DES exposure may cause CCA remains unclear. It is suggested by some authors that DES may inhibit the growth of anterior vaginal plate cells, which allows for the survival of glandular epithelium in the vagina in the form of adenosis (17, 18). Other hypotheses suggest that DES may act as a promoter-requiring co-carcinogen (17) or may induce DNA replication errors that remain unrepaired, resulting in microsatellite instabilities (19).

DES-naïve PCCAV has been reported to occur in patients with either vaginal adenosis (20) or genitourinary tract malformations (6). These were absent in our patient. Postmenopausal women have also been projected to be at an increased risk of developing non-DES-related PCCAV (9). In addition, other studies such as those by Haddout et al. and Thomas et al. report that CCAs also tend to affect older women (4, 21). Furthermore, PCCAV is believed to occur in patients with *in utero* exposure to DES at a younger age and at older ages in those without such exposure (22). However, this has not been proven in other studies looking at the development of PCCAV in patients with no *in utero* exposure to DES, as they found a bimodal age distribution in that population (17–19, 23). Some studies went further to qualify this bimodal age distribution as being associated with genitourinary abnormalities in younger women (17) and endometriosis in postmenopausal women (18). This bimodal age distribution with its associations as reported by Ikeda et al. and Shah et al. has not been replicated in other studies. For example, in the Netherlands, 41 PCCAV patients without *in utero* exposure to DES had their records from the central registry analyzed, and a bimodal age distribution with the first peak at 26 years and a second peak at 71 years was reported (19). A study by Jiang et al. also supported a bimodal age association with CCA in the post-DES period (23). These studies instead reported that this bimodal age distribution of non-DES-related CCA was associated with menarche and menopause (19, 24). Also, in a review by Chatterjee et al., 50% of the PCCAV patients with no *in utero* exposure to DES had no Mullerian abnormality or vaginal adenosis (5). It is also evident that non-DES-related PCCAV does occur in the younger population and may not be associated with genitourinary abnormalities or menarche per se, as there was a 9-year-old in the review by Chatterjee et al. (5), and we also present a 17-year-old with a case of non-DES-related PCCAV without these associations. The findings from these studies, including our case report, suggest the possibility of other risk factors that are currently unknown and may require further investigations.

Regardless of risk factors, the location of distant metastases from PCCAV is most often found in the lung, supraclavicular lymph nodes, and within the pelvis; although in rare instances, PCCAV can also spread to the liver, peritoneum, omentum, or ovaries (25). Such metastases may occur as long as 20 years after the resection or treatment of the primary vaginal tumor (3, 26, 27). Despite the propensity of PCCAV to metastasize beyond the pelvis, spread to the central nervous system is extremely uncommon. With that being said, there is one documented case of a primary cervical CCA that metastasized to the brain (3, 12). However, to the best of our knowledge, there have been no published cases of cerebral metastases from a non-DES-related PCCAV. We therefore present this case to highlight the possibility of non-DES-related PCCAV metastasizing to the brain, as well as potential unknown risk factors that may predispose women without *in utero* DES exposure to developing PCCAV. This will hopefully stimulate further discussion of the disease, contribute to the existing body of knowledge, and inform future management guidelines, including the role of neurosurgery in the management of these cases. This is important because, regardless of DES exposure, metastatic PCCAV is managed on an individual basis, as there are currently no established guidelines for its management because of its rarity (23). This gap is evident in the management of our patient. The ESTRO/ESGO/SIOPe guidelines for the management of patients with vaginal cancer published in 2023, outline management practices largely extrapolated from those with cervical cancers. It provides guidelines on the management of vaginal cancers, including those found in the pediatric and adolescent populations with vaginal rhabdomyosarcoma (RMS) and granulosa cell tumors/yolk sac tumors GCTs/YSTs but is silent on clear cell adenocarcinoma (28), probably due to its rarity.

## Conclusion

Non-DES-related PCCAV is extremely rare and carries an even poorer prognosis compared to DES-related PCCAV. We report a case of cerebral metastasis of PCCAV in a young female patient with no history of *in utero* exposure to DES and no genitourinary abnormalities. This case adds to the existing literature, highlights the need for further studies regarding risk factors for developing non-DES-related PCCAV, and showcases the role of neurosurgery in the management of these cases. The case also demonstrates the gap in management guidelines and advocates developing guidelines for the management of PCCAV.

## Data Availability

The original contributions presented in the study are included in the article/supplementary material. Further inquiries can be directed to the corresponding author.
